# What a Smile Means: Contextual Beliefs and Facial Emotion Expressions in a Non-verbal Zero-Sum Game

**DOI:** 10.3389/fpsyg.2016.00534

**Published:** 2016-04-19

**Authors:** Fábio P. Pádua Júnior, Paulo H. M. Prado, Scott S. Roeder, Eduardo B. Andrade

**Affiliations:** ^1^Programa de Pós-Graduação em Administração, Universidade Federal do ParanáCuritiba, Brazil; ^2^University of CaliforniaBerkeley, CA, USA; ^3^Fundação Getulio Vargas–Escola Brasileira de Administração Pública e de EmpresasRio de Janeiro, Brazil

**Keywords:** emotion, beliefs, interpersonal interaction, facial expression, sex, smile

## Abstract

Research into the authenticity of facial emotion expressions often focuses on the physical properties of the face while paying little attention to the role of beliefs in emotion perception. Further, the literature most often investigates *how* people express a pre-determined emotion rather than *what* facial emotion expressions people strategically choose to express. To fill these gaps, this paper proposes a non-verbal zero-sum game – the Face X Game – to assess the role of contextual beliefs and strategic displays of facial emotion expression in interpersonal interactions. This new research paradigm was used in a series of three studies, where two participants are asked to play the role of the sender (individual expressing emotional information on his/her face) or the observer (individual interpreting the meaning of that expression). Study 1 examines the outcome of the game with reference to the sex of the pair, where senders won more frequently when the pair was comprised of at least one female. Study 2 examines the strategic display of facial emotion expressions. The outcome of the game was again contingent upon the sex of the pair. Among female pairs, senders won the game more frequently, replicating the pattern of results from study 1. We also demonstrate that senders who strategically express an emotion *incongruent* with the valence of the event (e.g., smile after seeing a negative event) are able to mislead observers, who tend to hold a *congruent* belief about the meaning of the emotion expression. If sending an incongruent signal helps to explain why female senders win more frequently, it logically follows that female observers were more prone to hold a congruent, and therefore inaccurate, belief. This prospect implies that while female senders are willing and/or capable of displaying fake smiles, paired-female observers are not taking this into account. Study 3 investigates the role of contextual factors by manipulating female observers’ beliefs. When prompted to think in an incongruent manner, these observers significantly improve their performance in the game. These findings emphasize the role that contextual factors play in emotion perception—observers’ beliefs do indeed affect their judgments of facial emotion expressions.

## Introduction

Much research has been conducted to assess and improve people’s ability to distinguish authentic from inauthentic facial emotion expressions. This literature presents a few important characteristics. First, it most often focuses on the physical properties of the face (Ekman, 1992c); how these properties map onto basic or universally recognized emotion expressions (Ekman, 1992a; Tracy and Robins, 2008) as well as how they allow an observer to assess the authenticity of a facial emotion expression (Ekman et al., 1988; Ekman et al., 1990). The neuroscience literature has investigated the role of various brain structures in the perception and processing of emotions (e.g., Van den Stock et al., 2015) as well as how bodily expressions and other contextual cues affect facial perception and processing (see Van den Stock and de Gelder, 2014; Van den Stock et al., 2014a,b) but surprisingly, as it has been recently argued (Niedenthal et al., 2010; Barrett et al., 2011), the role that contextual factors play in emotion perception has nevertheless received relatively little attention, despite these factors clearly having an impact on how observers ultimately judge facial emotion expressions.

While expression authenticity can occasionally be detected from markers such as the Duchenne smile (see Ekman et al., 1988), these markers are not always reliable and context may provide additional useful authenticity cues (Hess and Hareli, 2015). What is more, empirical findings also suggest that a reliance on the physical expressions of the face alone is not sufficient to adequately recognize emotion when context is properly acknowledged and assessed. When an observer is asked to evaluate someone else’s emotion expression, the observer will normally rely also on contextual cues in order to detect the emotional information expressed on the sender’s face (Barrett et al., 2011). Individuals may interpret the meaning of an emotional expression in terms of the specific situations, events and behaviors that they know to be associated with that expression. For instance, a person might hold the belief that smiles are likely inauthentic during a sales interaction, but genuine if they occur as a result of a successful sale (Maringer et al., 2011). In similar fashion, anger is more likely to be perceived as fear when the angry facial expression is displayed within a frightening context, and sadness is more likely to be interpreted as disgust when embedded in a disgusting context (Carroll and Russell, 1996).

Second, this literature has often relied on relatively contrived procedures. This is particularly notable when the research questions focus on the differences between genuine vs. fake expressions. In these settings, participants are either instructed to display a given emotional reaction to specific (often neutral) stimuli or are simply observed displaying a spontaneous facial expression after exposure to an emotional stimulus. Then, the (in)authentic expressions are videotaped and coded—or observed by other participants—in search of differences in the physical properties of the face across conditions (Ekman et al., 1988; Hess and Kleck, 1990; Smith et al., 1996; Krumhuber and Manstead, 2009). This paradigm allows the sender to decide, for instance, how to exhibit a fake smile after seeing a neutral stimulus. However, it does not help them decide whether to smile, frown or maintain a neutral expression. That is, it leaves little room for participants to strategically decide which signal to send, if any. The paradigm is also most often one-sided. Given the absence of an online dyadic interaction, senders have no need to draw inferences about the observer’s beliefs regarding the meaning of the sender’s own facial expressions, which could in turn impact the sender’s decision about which emotion to display in the first place.

Finally, it is well-established that observers often do slightly better than chance when assessing the truthfulness of a sender’s signal (i.e., the person who expresses the emotion; Kraut, 1980; Vrij, 2000; Bond and De Paulo, 2006), though this effect tends to weaken when the sender’s signal comes from facial expression only relative to facial and verbal cues combined (Bond and De Paulo, 2006). Nonetheless, observers attempting to guess the authenticity of a sender’s signal rarely perform *worse* than chance. Put simply, a review of extant literature suggests that senders are unlikely to ‘outsmart’ observers.

This paper presents a new research paradigm—a non-verbal zero-sum game—to test the role of contextual factors (e.g., beliefs about the meaning of a facial emotion expression) and strategic displays of facial emotion expressions in dyadic interactions. It also assesses whether or not conditions exist under which senders can systematically perform better than observers.

Given that this game is relatively novel, we adopt an inductive, exploratory approach for study 1. That is, no specific hypothesis is elaborated *a priori*. Once a given pattern of results is obtained, subsequent confirmatory studies will assess its reliability and underlying process.

## Materials and Methods – Overview

This paper proposes a new research paradigm—a non-verbal zero-sum game called the *Face X Game*—to assess the role of contextual beliefs and strategic displays of facial emotion expressions in interpersonal interactions.

Since participants must “stare” at each other in a competitive setting, this game has the potential to be slightly uncomfortable when played with strangers. As a consequence, throughout all three studies, care has been taken to perform each experimental session in an environment where most participants were of similar age and at least acquaintances (i.e., classmates). Thereafter, the sample was comprised mostly of classmates. The volunteers were invited to participate in this study in exchange for course credit and monetary payment (based on task performance). Prior to each experimental session, tables and chairs in the classroom were arranged in pairs so participants could be placed face-to-face. A set containing instructions for the game, a player and observer’s sheet, as well as a player and observer’s final questionnaire was placed on each table. Each set was numbered such that even numbers were assigned to observers and odd to players. Each pair was comprised of sequential numbers (e.g., 1 and 2; 3 and 4; and so on). After this initial preparation, participants were allowed to enter into the classroom, one at a time. Next, each participant randomly selected a numbered piece of paper from a bag held by the experimenter. They were then instructed to sit at the table corresponding to the number on the paper. This exercise assigned them to their role (i.e., player or observer). Each session lasted 30 min and was conducted in classrooms with space for 10–15 pairs. Care was taken to avoid participants that had either already played the game or even simply heard of it. Once all the participants were seated, the experimenter asked them to put away anything they had brought with them (apart from a pen), and told them to read the instructions in front of them on their tables. Consent forms were then signed. Participants explicitly agreed to participate and to confirm that they had never been to the same study or similar studies in the past 6 months. Then, the experimenter read the following text out loud: “This is an experiment on decision making. The instructions are simple. If you follow the rules carefully and make good decisions, you can earn a considerable amount of money that will be paid in cash at the end of the experiment. Different participants can earn different amounts of money. What you earn today depends in part on your decisions, in part on the decisions of others and in part on luck. It is important that you do not look to the decisions of others, you do not talk or make loud noises, and you strictly follow the task instructions. You will be advised if you violate the rules the first time. If you violate it a second time, you will be asked to leave the room and you will not receive payment. So read the rules in the instruction sheet and wait for the signal to start the game.”

Before handing out the cards for the trial, the examiner additionally explained all details and steps of the game, reminding participants to avoid showing their notes to their partners throughout the game. Participants were instructed from that moment on to avoid talking to their partners.

The Face X Game is very simple. In this task two participants are matched at random, placed face-to-face, and asked to play the role of the player^1^ or the observer. Two cards are distributed per pair and sit face down on the table between them. When flipped, each card has either a currency symbol ($) or a zero (0) on its face, which indicate “money” vs. “no money” respectively (hereafter card$ and card0). The player’s main task is to slowly, sequentially look at each card before returning it face down to the table. The observer’s main task is to choose one of the two cards. Both players are informed that the purpose of the game is to determine the extent to which the observer is capable of guessing which of the two cards has money on it (i.e., which card is card$) solely by observing the player’s facial expression during and after s/he looks at each of the two cards. Any verbal communication is strictly prohibited. Recall that this is a zero-sum game. If the observer correctly chooses card$, the observer wins. Otherwise, the player wins. It is worth noting that, in order to encourage participants to act naturally, instructions made no mention of facial expressions nor gave any indication that players might try to deceive observers. In addition, despite having already received a questionnaire, participants were told not to read it until the end of the game.

In order to test participants’ understanding of the game, a moneyless preliminary practice round precedes the actual task. No feedback is provided during this trial—that is, participants are not made aware of their performance. Both parties take notes about the card that is seen by the player, as well as the observer’s choice, but they are not allowed to exchange this information with each other. The examiner does not verify this preliminary round’s outcome since no money is involved. The purpose of the trial is simply to familiarize participants with the procedure rather than provide an opportunity for the player to adjust his or her behavior in the light of the observer’s response. After the practice round is completed and all questions about the procedure are answered, the experimenter places a R$10 bill (exchange rate of Brazilian currency at the time: R$1.00 ≈ U$0.50) on each pair’s table, and the actual game begins. All participants have complete information about the rules of the game. The game progresses in the following manner: First, the examiner gives a signal and the player looks at card 1 to check if it is card$ or card0. The player must look at the observer (eye-to-eye) for a few seconds before he puts the card back on the table. The observer then takes the pen from the table, indicates on the observer’s sheet his or her guess as to which card it might be and puts the pen back down. The observer’s options are (1) I am certain this is the $10 Card, (2) I think this is the $10 Card, (3) I have no idea, (4) I think this is the $0 Card, or (5) I am certain this is the $0 Card. At the same time, the player takes the pen, indicates on the player’s sheet whether card1 was card$ or card0, and ends the round by placing the pen back on the table. The same process is repeated for the second card (see Supplementary Material for a more detailed description of the procedure). Note that the observer forms his or her impression of each card and reports it, after both rounds. At the end of the second actual round (i.e., after card 2), the observer must then indicate which of the cards s/he believes has the $10 printed on it. If s/he guesses correctly, s/he receives the R$10 bill. If s/he gets it wrong, his or her partner will receive the R$10 bill. Only one choice is allowed for the final choice. The main purposes of the initial assessments were to (a) force participants to form an impression based on the facial expression after each card and (b) keep a constant flow to the game. Observers are aware that only the final choice matters. At the end of the game, both participants are asked to fill out a questionnaire and told that only complete questionnaires will be accepted. Note that all participants begin each of their tasks throughout the game at the same time and in accordance with the examiner’s signal to begin. They are able to see the outcome of the game only after completing this final task. To ensure accuracy, the experimenter additionally double-checks the outcome of the game for each dyad. In all three studies conducted winners actually receive R$10.00 whereas losers leave with $0.

All three studies took place in two cities. Among 217 pairs, 30 (13.8%) were from Berkeley, California (USA) and 187 (86.2%) were from Curitiba, Paraná (Brazil). Participants in Brazil received a R$10 bill whereas those in the United States were given a US$10 bill. While it is possible that culture differences exist between Brazil and the United States, care was taken to ensure that there were no noticeable dissimilarities in the materials, procedure or instruction sets. Importantly, no differences in the outcome of the game were detected between locations.

Conceptually, this task presents three unique characteristics compared to past tasks conducted in the literature. First, the player can strategically choose which facial expression to display and how to display it after each positive (card$) or negative (card0) event. Second, the observer’s beliefs about the likelihood that the player will display a given facial expression (e.g., smile after seeing card$) is expected to impact the observer’s card choice. Finally, the dyadic and online face-to-face nature of the task also prompts the player to make a guess as to the observer’s beliefs in an attempt to ‘outsmart’ the partner, and vice-versa.

The following three studies use the Face X Game to assess the role of strategic facial emotion expression and contextual factors on dyadic interactions. Study 1 focuses on the outcome of the game itself, study 2 examines the strategic display of facial emotion expressions, and study 3 investigates the role of contextual factors. Exact procedures used in each of the studies can be found in Supplementary Material.

### Study 1

Study 1 examined the outcome of the game with reference to the sex of the pair.

#### Participants

One hundred sixty-four undergraduate students (40.4% female; mean age = 23.3, *SD* = 4.68) participated in this study in exchange for course credit and monetary payment (based on task performance). Prior to the beginning of the study all participants were given a consent form to read and sign (pending agreement). The study was approved by the Ethics Research Committee CEP/SD at Federal University of Paraná, Brazil, and the Committee for Protection of Human Subjects at the University of California, Berkeley, CA, USA.

#### Procedure

Participants in each experimental session were randomly paired, assigned to either the role of player or observer, and instructed to play the Face X Game. In each pair, one participant ended the game with R$10.00 whereas the other earned nothing. At the end of the game, all participants completed a short survey. They were asked about their expected outcome ($0 or $10); the strategy they used to maximize their payoff (open-ended question); perceived level of friendship with their paired-partner (nine-point scale ranging from 0 = I have never talked to this person before to 8 = I interact with this person on a daily basis) and experience as a poker player (1 = never/rarely; 2 = sometimes; 3 = always). We also gathered information about their personality traits and their affective states during the game. Besides these control variables, our main dependent variable is the winner of the game and the independent variable is the pair’s sex mix.

#### Results and Discussion

The *p*-value cutoff used in all statistical analysis throughout this work is *p* = 0.05. Following the recommendation of some statisticians, we did not correct for multiple comparisons while analyzing data, since we report all of the individual *p*-values (Rothman, 1990; Saville, 1990).

Statistical analyses uncovered two intriguing findings. First, out of the 82 pairs, observers only won the game 31 times. Put another way, 62.2% of players were able to mislead their observers. A z-test revealed a statistically significant difference of players’ wins compared to chance (*z* = 2.21, *p* < 0.05). A Chi-square test showed that this effect was not contingent upon whether the first card seen in the practice or actual game was card$ or card0 [practice game: χ^2^(1, *N* = 82) = 2.02, *p* > 0.10; actual game: χ^2^(1, *N* = 82) = 1.17, *p* > 0.10]. In short, contrary to the bulk of findings in the emotion expression literature demonstrating that observers perform slightly better than chance on average (Kraut, 1980; Vrij, 2000; Bond and De Paulo, 2006), our first study shows that *players* were more likely to win at this non-verbal zero-sum game.

Second, a Chi-square test revealed that the outcome of the game varied significantly by sex-pair [χ^2^(3, *N* = 80) = 12.31, *p* = 0.006]. Players won more frequently when the pair was comprised of at least one female. The only condition in which observers were more frequent winners was when two men played against each another (see **Table 1**-study 1).

**Table 1:** Frequency and percentage of winners in the Face X Game across the studies.

	Winner	
	Player	Observer
Study 1, *n* (%)^1^		
F-F	9 (75.0)	3 (25.0)
M-M	12 (38.7)	19 (61.3)
FO-MP	16 (80.0)	4 (20.0)
FP-MO	13 (76.5)	4 (23.5)
Study 2, *n* (%)		
F-F	30 (68.2)	14 (31.8)
M-M	16 (45.7)	19 (54.3)
Study 3, *n* (%)^2^		
F-F with congruent beliefs	16 (61.5)	10 (38.5)
F-F with incongruent beliefs	9 (32.1)	19 (67.9)

*^1^Two participants did not report their sex in study 1. F-F, female player and observer; M-M, male player and observer; FO-MP, female observer and male player; FP-MO, female player and male observer. ^2^Two participants provided answers that were both congruent and incongruent (e.g., $ means smile and 0 means smile). They were deleted from the analysis.*

Assuming that performance is at least somewhat related to the facial emotion expressions displayed (since it is a non-verbal procedure), it is possible that individual variation in facial expressions led to the observed results. However, this does not explain either the overall player’s dominance or sex interaction.

Judging a smile as genuine is the normative judgment. A true smile denotes positive feelings or intentions (Niedenthal et al., 2010). Thus, it is likely that an observer, regardless of sex, will tend to believe that a smile means that the player has received a stimulus with positive valence (card$). One may argue that, since this is a competitive game and not representative of a naturalistic situation, observers might simply adjust their beliefs and start guessing that smiles indicate an attempted deception. However, as mentioned above, participants were given no indication that players would be trying to deceive observers via facial expressions. We therefore argue that observers have kept their prior belief, i.e., a smile means that a player has seen card$. What is more, women tend to encode facial expressions of emotion better than men (Fujita et al., 1980), and smile more than men (Haviland, 1977; Hall, 1984; Hall and Halberstadt, 1986; Burgoon et al., 1989; LaFrance et al., 2003; Ellis, 2006). This could explain in part why female players had such an advantage over observers. For example, female players may have also smiled after receiving a stimulus with negative valence. Whereas observers (both male and female) might hold on to their contextual beliefs that smiles are the representation of a stimulus with positive valence, female players in this task were able to systematically mislead observers.

If we suppose that smiles were a key factor in explaining the players’ outcome, the results of this first study indicate that players probably smiled more in all pairs that included at least one woman. Since the lone condition where observers won more frequently (61.3%) were male-only pairs, it is possible that male players simply smiled less in that condition. Several studies indicate that men do smile less in the presence of other men (LaFrance et al., 2003). One possible explanation for this behavior is that smiling norms are more apparent when people interact with partners of the same sex. Men smile less in the presence of other men because any expression that does not include a smile is often classified as more dominant than an expression containing a smile (Keating, 1985). Consequently, male players in our study may have engaged in dominance-seeking in the presence of male observers, which caused them to smile less under these conditions. However, in the presence of female observers, it is likely that this attitude changes, causing male players to smile more compared to when they are paired with male observers. This could explain, in part, the apparent advantage obtained by male players (80% win) compared to when they are paired with female observers.

A short survey was given at the end that sought game-related information (e.g., expected outcome, strategy used to maximize payoff, level of friendship, experience as a poker player, as well as personality traits and affective state). None of these control variables had any impact on the outcome of the game in study 1.

We did not collect observers’ anecdotal assessments of players’ facial expressions in study 1. Therefore, it is impossible to conclusively determine whether smiles indeed varied by condition. Further, given the sample size per sex-pair, it is worth further assessing the robustness of these findings. Study 2 tackles the issues above. Moreover, in the second study, we investigate the possibility that players who have smiled more often after seeing card0 end up deceiving observers (who may believe that a smile means the player saw card$ in that context).

### Study 2

The purpose of study 2 is twofold. First, it tests the robustness of the findings observed in study 1 for same-sex pairs. We focus on same-sex pairs because that is where differences in emotion expression, and particularly smiles, are most pronounced. In an extensive meta-analysis, LaFrance et al. (2003) showed that the effect size for same-sex pairs on smiling was significantly larger than for opposite-sex pairs. Second, we assess perceived differences in facial emotion expressions across these two groups by directly asking observers to report on the players’ facial expressions. Observational evidence from study 1 indicated that players’ expressions varied from very serious to smiley, both while looking at the cards as well as after, with a significant portion of them maintaining what could be anecdotally described as a “poker face” (i.e., neutral expression). We therefore asked participants in study 2 to judge the players’ expression on a scale from very serious to very smiley (see Procedure for more details of this scale). To assess whether different expressions also alter the perceived authenticity of the expression, observers were also asked to judge the genuineness of the player’s expression.

#### Participants

One hundred fifty-eight undergraduate students (55.7% female; mean age = 27.5, *SD* = 5.14) participated in this study in exchange for course credit and monetary payment (based on task performance). Prior to the beginning of the study all participants were given a consent form to read and sign (pending agreement). The study was approved by the Ethics Research Committee CEP/SD at Federal University of Paraná, Brazil, and the Committee for Protection of Human Subjects at the University of California, Berkeley, CA, USA.

#### Procedure

The procedure closely paralleled study 1. Participants were randomly paired, assigned to their roles as player or observer, and instructed to the play the Face X Game. Study 1 and study 2 differed in two primary ways. First, in this study, only same-sex pairs were formed. Second, *after* observers had made their card choice (the main DV), they were asked to indicate the expressed emotional valence of the player after the player had seen the first and second cards on two seven-point scales (one for each card): from -3 (Serious) to +3 (Smiley), with a midpoint 0 = Neutral. As mentioned above, observers were also asked to indicate on two seven-point scales (one scale for each card) the perceived authenticity of the expression of the player after the player had seen the first and second cards: from -3 (Clearly fake) to +3 (Clearly genuine), with a midpoint 0 = Can’t tell. To keep the flow identical during and after the game to both players and observers, we also asked players to report what emotion they thought they had expressed, as well as how genuine they thought their expression appeared to be to observers. The same four seven-point scales were used. At the end of the game, all participants filled out a questionnaire. Some of the questions were the same as study 1: expected outcome ($0 or $10); the strategy they used to maximize their payoff (open-ended question); perceived level of friendship with their paired-partner (nine-point scale ranging from 0 = I have never talked to this person before to 8 = I interact with this person on a daily basis) and experience as a poker player (1 = never/rarely; 2 = sometimes; 3 = always). Two new questions were inserted: Their preferred role if they could choose (i.e., player or observer) and the extent to which they cared about the money at stake (nine-point scale ranging from 0 = I do not care at all to 8 = I care a lot). Once again, our dependent variable is the winner of the game and the independent variable is the pair’s sex mix. The mediator variable is the (in)congruency index.

#### Results

##### Outcome of the game

A Chi-square test revealed that the outcome of the game was again contingent upon the sex of the pair [χ^2^(1, *N* = 79) = 4.05, *p* = 0.044]. When female players and observers played against one another, players won the game more frequently, replicating the pattern of results from study 1. A z-test revealed a statistically significant difference of players’ wins (68.2%) compared to chance (*z* = 2.41, *p* < 0.01). Among male pairs, however, players won only 45.7% of the time. A z-test indicated no significant difference of players’ wins compared to chance (*z* = -0.50, *p* > 0.10; see **Table 1**-study 2).

##### Emotion expression

**FIGURE 1:**
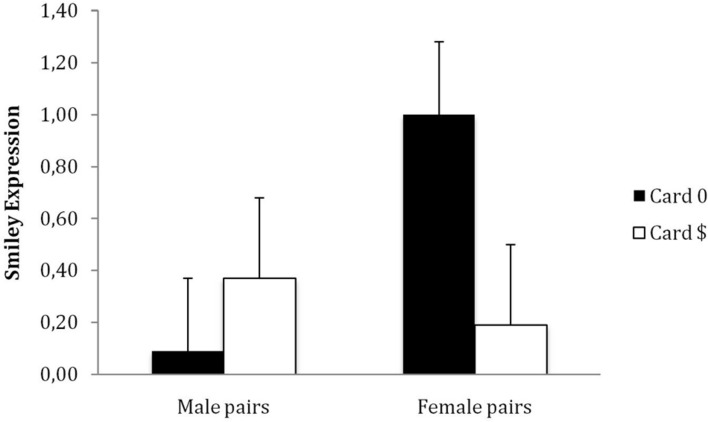
**Player’s facial emotion expression (as reported by the observers) – study 2.** Error bars represent standard errors.

A GLM Univariate Analysis was conducted to explore if the congruency of facial emotion expression varies by the sex of the pair (male vs. female) and by card (card0 vs. card$). The players’ emotion expressions as judged by the observers varied marginally by card and sex [*F*(1,152) = 3.42, *p* = 0.07; $\eta_{p}^{2}$ = 0.02]. Among female pairs, players were more prone to display an *incongruent* expression—to smile more after seeing card0 than after seeing card$ [*M*_0_ = 1.00, *SD* = 1.83 vs. *M*_$_ = 0.19, *SD* = 1.94; *F*(1,152) = 4.17, *p* = 0.049; $\eta_{p}^{2}$ = 0.03]. Among male pairs, however, there was no difference between these two means. Male players seemed more inclined to display a congruent expression—that is, to smile slightly more after seeing card$ (*M*_0_ = 0.09, *SD* = 1.72 vs. *M*_$_ = 0.37, *SD* = 1.86; *F* < 1). Female players were also more inclined to smile after card0 than males players [*F*(1,152) = 4.72, *p* = 0.028; $\eta_{p}^{2}$ = 0.03] – See **Figure 1**. It is worth noting that the players’ self-assessed facial emotion expressions were collected. The relationship between the observers’ perception of the players’ facial expression and the players’ perception of their own facial expression was investigated using Pearson product-moment correlation coefficient. Preliminary analyses were performed to ensure no violation of the assumptions of normality, linearity and homoscedasticity. There was a strong, positive correlation between the two variables, *r* = 0.50, *p* < 0.0001.

##### Authenticity on the outcome of the game

There was no interaction between card content and sex on the perceived authenticity of the expression and none of the main effects were significant (*F*s < 1). These null effects suggest, for instance, that independent of whether the player had seen a positive (card$) or a negative (card0) event, observers saw similar levels of authenticity in both male and females’ smiles.

##### Emotion expression on the outcome of the game

An index was created to assess congruency or incongruency between the valence of the card and the emotion expression of the observer. The index was calculated as the sum of the intensity of the player’s (in)congruent expressions reported by the observer throughout the game (-6 = maximal incongruency to +6 = maximal congruency). For example, a player who displayed a level 3 smile after card0 and a level 2 seriousness after card$ would score -5 on the index, whereas a player who displayed a level 1 seriousness after card0 and a level 2 smile after card$ would be categorized as +3 on the same index. Note also that a player who displayed the very same expression after seeing both cards (whatever the expression), would land on “0” on the (in)congruency index.

**FIGURE 2:**
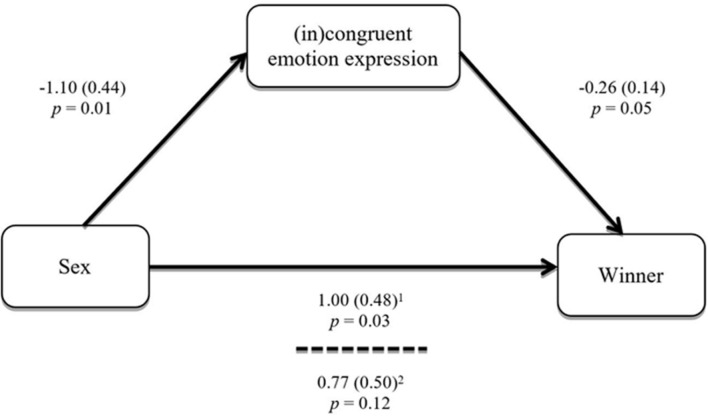
**Mediation analysis – study 2.** Statistics – coefficient (SE), *p*-value. Coding – *Sex*: 1 = female, 0 = male; *Facial Emotion Expression:*-6 = maximum incongruency, +6 = maximum congruency; *Winner*: 1 = player; 0 = observer. Normal theory test is not allowed for models with dichotomous outcomes (Preacher and Hayes, 2008). ^1^Total effect (c path); ^2^Direct effect (c’ path).

Results from a one-way analysis of variance were consistent with previous analyses, indicating that female players showed higher levels of incongruency (*M* = -0.81; *SD* = 1.91) relative to male players [*M* = 0.29; *SD* = 1.99; *F*(1,76) = 6.16, *p* = 0.01], who, on average, displayed only slightly congruent expressions during the game. A one-sample *t*-test was conducted to compare the incongruency scores with zero in each group. There was a significant difference from zero in scores for female pairs [*M* = -0.81, *SD* = 1.91; *t*(42) = -2.80, *p* = 0.01, two-tailed]. The magnitude of the differences in the means (mean difference = -0.814, 95% CI: -1.40 to -0.23) was very large (η^2^ = 0.16). For male pairs, there was no significant difference from zero in incongruency scores [*M* = 0.29, *SD* = 1.99; *t*(34) = 0.85, *p* = 0.29, two-tailed]. Of importance, a mediation analysis was also conducted to assess whether this difference in congruency could at least in part explain the impact of sex on the outcome of the game. Following Preacher and Hayes (2008), an INDIRECT test was conducted with sex as the IV (1 = female; 0 = male), the (in)congruency index of emotion expression as the mediator (-6 = maximum incongruency; +6 = maximum congruency), and the outcome of the game as the DV (winner: 1 = player; 0 = observer). The relationship between sex of the pair and winner of the game was mediated by the (in)congruency index of the emotion expression (i.e., players’ facial emotion expressions). As **Figure 2** illustrates, results indicated that sex was a significant predictor of the players’ facial emotion expressions, *b* = -1.10, *SE* = 0.44, *p* = 0.01, and that the players’ facial emotion expressions were a significant predictor of who won the game, *b* = -0.26, *SE* = 0.14, *p* = 0.05. In this study, the sex of the pair was no longer a significant predictor of winner of the game after controlling for the mediator, *b* = 0.77, *SE* = 0.50, *p* = 0.12, consistent with full mediation. The indirect effect was tested using a bootstrap estimation approach with 1000 samples. Results from this test indicated the indirect coefficient was significant, *b* = 0.314, *SE* = 0.223, 95% CI = 0.0274, 0.9417. In sum, the direct impact of sex on the outcome of the game was mediated by the players’ facial emotion expression during the game. Female players were more likely to express a signal that was incongruent with the card, which in turn increased their chance of winning the game.

Another mediation analysis was performed with sex as the IV, the perceived authenticity as the mediator, and the outcome of the game as the DV. In line with the pattern obtained previously, results indicated that perceived authenticity did not mediate the impact of sex on the outcome of the game. In other words, the observer’s perception of the player’s expression did not affect the final result of the game.

#### Discussion

Study 2 replicates study 1’s results with regards to sex-pair, which once again significantly impacted the outcome of the game. Among male pairs, observers still seem to possess a small advantage over players, consistent with the literature (Ekman and O’Sullivan, 1991; Ekman, 1992b; Ekman et al., 1999; Bond and De Paulo, 2006; Bernstein et al., 2008; Porter and Brinke, 2008). This trend was, however, non-significant. More interestingly, and in line with study 1, players performed significantly better than observers among female pairs. These results seem to rely on female players’ tendency to display incongruent emotion expressions (e.g., smiling after seeing card0).

Overall, this study’s findings are in harmony with several researches showing that women are significantly more likely to smile in an inauthentic manner – as well as use these fake smiles to mask negative emotions – than men (Prkachin and Silverman, 2002; LaFrance et al., 2003; Woodzicka, 2008). Despite these observations, facial expressions (enjoyment smiles, non-enjoyment smiles, and neutral expressions) from female players are generally perceived as more approachable (Miles, 2009) and women may be perceived more favorably when smiling (Hack, 2014). It is worth noting that women typically smile more often than men when social tension is high (Hall and Halberstadt, 1986). With reference to the above, it is possible that participants in this task did feel some degree of tension while staring at their partner. This tension might then help to explain why women smiled more than men, particularly after having seen card0. Finally, the literature suggests that smiling could be overall more beneficial for women (Mehu et al., 2008). For men, smiles and facial expressions seem to be less positively received than for women (Fujita et al., 1980).

Although, rating facial expressions on a Likert scale rather than objective assessors or video recording may be considered a limitation, for the purpose of this study it is quite useful. We decided *a priori* that we were not interested in measuring complex facial expressions nor micro expressions since the presence of this type of equipment might distract participants, add uncontrolled variables, make them aware that their facial expressions could indeed play a major role in the outcome of the game, or otherwise generally cause them to alter their behavior. Instead, we decided to measure only three simple facial expressions—enough to assess the congruency or incongruency between the valence of the card and the emotion expression of the observer. These expressions were ‘smiley,’ ‘neutral’ (poker face), and ‘serious’ (the opposite of ‘smiley’).

Similar to study 1, none of the control variables (expected outcome; strategy used to maximize payoff; level of friendship; experience as a poker player; preferred role; attitude toward the money at stake) impacted the outcome of the game in study 2. After study 1, we stopped asking about personality traits and affective states, simply because the questions added unnecessary length to the experiment and, more importantly, did not impact the results.

If sending an *incongruent* signal (i.e., smiling after card0) helps to explain why female players win more frequently, it logically follows that female observers were more prone to hold a *congruent*, and therefore inaccurate, belief (e.g., “if the player smiles, it means that she has seen card$”). This prospect seems quite interesting. It implies that while female players are willing and/or capable of displaying fake smiles, paired-female observers are not taking this into account. Study 3 tackles this issue by manipulating female observers’ beliefs.

### Study 3

Only female pairs were recruited for study 3. To test the role of beliefs, female observers were prompted to form either congruent or incongruent beliefs about the meaning of a given facial emotion expression. Some were led to form a belief that players tend to frown after seeing the card0 and to smile after seeing card$ (i.e., a congruent belief), whereas others were led to form incongruent beliefs. The impact of formed beliefs on the outcome of the game was then assessed. It was expected that when thinking in an incongruent manner, which is most often the accurate way of thinking, female observers would significantly improve their performance.

#### Participants

One hundred twelve female undergraduate students (mean age = 21.7, *SD* = 3.27) participated in this study in exchange for course credit and monetary payment (based on task performance). Prior to the beginning of the study all participants were given a consent form to read and sign (pending agreement). The study was approved by the Ethics Research Committee CEP/SD at Federal University of Paraná, Brazil, and the Committee for Protection of Human Subjects at the University of California, Berkeley.

#### Procedure

Similar to study 2, participants were randomly paired, assigned to their roles, and instructed to the play the Face X Game. However, a main deviation from the previous procedure was implemented. After practicing, but before playing the actual game, observers were prompted to respond to three questions regarding their beliefs about what their paired-player’s serious vs. smiley expression might mean. For example, participants read: “In your opinion, what does it mean when a player smiles while/after looking at a given card?” An ostensibly “correct” answer was positioned at the lower right corner after each question was introduced in an attempt to persuade participants into forming either a congruent belief (i.e., a smiley (serious) face means a $ (0) sign on the card) or an incongruent belief (i.e., a smiley (serious) face means a 0 ($) sign on the card)—see Supplementary Material for complete details. We used the same procedure as the previous studies with regards to the assignment of roles (i.e., a numbered piece of paper selected from a bag held by the experimenter), observers were randomly assigned to either the congruent belief induction or to the incongruent belief induction, with half in each condition. The difference between these groups was how the “correct” answer on the observer’s sheet was written. Questions 1 and 2 discussed the potential meaning of smiles and serious expressions, respectively, while question 3 contained no manipulation. This last question was included to ensure that participants would differentiate serious from neutral expressions. If, as had occurred in the previous studies, female observers lose because they hold congruent beliefs while female players express incongruent emotions, observers should be more likely to win the game after forming an incongruent, and therefore more likely accurate, belief. It is also important to note that players were not explicitly aware of this manipulation. The procedure regarding the player was identical to the one used in the standard Face X Game. At the end of the game, all participants filled out a questionnaire that contained the same questions as study 2. Our dependent variable is the winner of the game and the independent variable is the contextual belief (congruent vs. incongruent belief).

#### Results

The belief induction worked as expected. A Chi-square test revealed that when prompted to form a congruent belief (i.e., when congruent belief answers appeared next to the questions), 63% of observers reported that a smile meant card$ and a serious face meant card0, whereas when prompted to form an incongruent belief (i.e., when incongruent belief answers appeared next to the questions), only 31% formed congruent beliefs [χ^2^(2, *N* = 56) = 9.54, *p* = 0.008].

Critical to our hypothesis, the observer’s beliefs had a significant impact on the observer’s likelihood of winning. A Chi-square test indicated that observers who held a congruent belief won the Face X Game only 38.5% of the time, whereas those who held an incongruent belief won 67.9% of the time [χ^2^(1, *N* = 54) = 4.69, *p* = 0.030; see **Table 1**-study 3].

Since we also gathered information about perceived facial expressions, we were able to assess the extent to which the observer’s belief and the (in)congruency index interacted on the outcome of the game. A logistic regression was conducted where the outcome of the game (0 = observer winner; 1 = player winner) was regressed on the observer’s belief (0 = congruent; 1 = incongruent), the (in)congruency index (-6 = maximal incongruent signal; +6 = maximal congruent signal), and the interaction term. The omnibus test of the model was significant [χ^2^(3) = 10.38, *p* = 0.016]. As already demonstrated, there was a main effect of belief, such that observers were more likely to win when they held an incongruent belief (*b* = 1.272, *SE* = 0.61, *p* = 0.037). Further, there was a significant interaction between the (in)congruency index and the observer’s beliefs on the outcome of the game (*b* = -0.87, *SE* = 0.41, *p* = 0.042). For observers who held a congruent belief, the more incongruent the expression of the player (e.g., smiling after seeing card0), the higher the likelihood that the *player* would win the game. For observers who held an *in*congruent belief, however, the effect reversed—the more incongruent the expression of the player, the higher the likelihood that the *observer* would win the game.

#### Discussion

Results from study 3 show that female observers were more prone to hold a *congruent* belief, which lessened their chances of winning the game. When prompted to think in an *incongruent* manner, their performance significantly improved. These findings emphasize the role of contextual factors on emotion perception—observers’ beliefs do indeed affect their judgment about facial emotion expressions (see also Carroll and Russell, 1996; Niedenthal et al., 2010; Barrett et al., 2011; Maringer et al., 2011).

It is worth noting that 63% of observers in the congruent bias induction actually formed a congruent belief. The ideal situation would be 100% of that group of observers. In this ideal scenario, one could expect a strengthening of the rate of players’ success. Since we did not reach that ideal condition, our results show that in study 3 players won a little less often (61.5%) than in the comparable pairs in study 1 (75%) and study 2 (68.2%). However, what is relevant is that players won more frequently than observers in the congruent belief condition, as expected.

At the end of the game, all participants completed a short survey containing the same questions as the previous study: the expected outcome; the strategy they used to maximize their payoff; perceived level of friendship with their paired-partner; experience as a poker player; preferred role and attitude toward the money at stake. As expected, none of these control variables impacted the outcome of the game in study 3.

## General Discussion

We have presented a novel paradigm, the Face X Game, to test the role of beliefs and strategic displays of facial emotion expression in interpersonal interactions. This paper also shows some of the conditions under which players perform better than observers. Unlike past research, the Face X Game allows players to freely choose their facial expressions after seeing each card, and observers are able to form their own beliefs about the meaning of those expressions. That is, they can decide for themselves whether a smile is the result of either a positive or negative event. Results from three studies show that female players often express an emotion *incongruent* with the valence of the event (e.g., to smile after seeing a negative event) and, as a consequence, can systematically mislead observers, who tend to hold a *congruent* belief about the meaning of this emotion expression (e.g., a smile signals a positive event). When prompted to think in an incongruent manner, female observers significantly improve their performance in the game. In a recent article, Schlicht et al. (2010) showed that people made more mistakes in a simplified version of Texas Hold’em when their virtual opponents’ facial expression was positive (vs. threatening). Similarly, our findings suggest that “observers” seem also more likely to hold a congruent belief – higher folding rates after perceiving a positive emotional display on their opponent’s face.

This work naturally has some caveats and, as a result, some interesting avenues for future research. First, no truly objective measures of participants’ facial expressions were recorded. It is possible that players relied on other cues, whether consciously or unconsciously, to conceal the truth. The same logic might apply to observers in their attempt to interpret the meaning of the player’s expression. While not measured, eye gaze might also play an important role. All of the studies’ tasks were synchronized by the experimenter, which means that the duration of eye gaze should have been nearly the same for all participants. However, a video recording of these interactions could provide useful information about how facial information is processed by observers. In short, we concede that videotaping participants while they play the game might help to address these concerns, but with the following caveat: As mentioned above, the act of being videotaped could itself alter participants’ behavior and add additional noise.

The fact that sex affected results is, at least in hindsight, not a complete surprise. The experimental economics (Croson and Gneezy, 2009; Mussel et al., 2014) and emotion expression literatures (LaFrance et al., 2003) often observe sex as a key moderator. However, we are left to only speculate as to the precise reasons that led to sex differences in emotion expressions in the Face X Game. One possibility may rely on people’s propensity to express a particular emotion in a given context and the ease to implement a given strategy. For instance, females appear to be more likely to smile than males generally, and it is possible that men are more likely to smile at women than at other men. If true, this tendency may lead to the implementation of an incongruent strategy in the right conditions (e.g., smile after seeing card0). Another factor may be differences in *level-k* thinking within and across pairs (Camerer et al., 2004). Note, however, that the game prevents us from making direct claims about the participants’ thought processes. For instance, we do not know whether an observer who appears to hold a congruent belief (e.g., a smile means that s/he holds card$) is being “too naïve” (level-0) or “too smart” (level-2). In either case, s/he would likely lose the game if the player adopted a level-1 strategy. Given the nature of the task, any mismatch between the player’s strategy and the observer’s beliefs will benefit the former (e.g., player level-0 or 2 and observer level-1 or 3). It requires a match between the player’s strategy and the observer’s beliefs to render the observer the winner (e.g., player level-0 or 2 and observer level-0 or 2; player level-1 or 3 and observer level-1 or 3).

As previously mentioned, across all three studies, a short survey at the end of each experimental session sought information not only about participants’ sex, but also age, level of friendship with their paired-partner, preferred role, expected outcome, experience as a poker player, and the extent to which they cared about the money at stake. Apart from sex, none of these other variables consistently impacted the outcome of the game. It is possible that a larger variance in the sample may lead some of these items to interact with the outcome of the game (e.g., age discrepancy or level of friendship). **Table 2** offers a descriptive snapshot of the samples’ characteristics across the studies. Lastly, it remains possible that other untested variables might also conform to this pattern of results. For example, would participants change their strategy if the money payout was ten times higher? Or, if there was no money at all? Did participants share their prize? If so, did this change their behavior during the game? These questions are interesting avenues to be addressed in future research.

**Table 2:** Descriptive characteristics of the samples across the studies.

	Study		
	1	2	3
Age –mean (SD)			
Player’s	23.4 (6.43)	27.8 (6.41)	21.6 (3.96)
Observer’s	23.3 (4.94)	27.2 (6.19)	21.8 (3.66)
Pair’s	23.3 (4.68)	27.5 (5.14)	21.7 (3.27)
Pair’s absolute age difference	3.52 (5.57)	5.67 (4.54)	2.64 (2.89)
Perceived friendship level –mean (SD) (0 = I’ve never talked to this person before; 8 = I interact with this person on a daily basis)			
Player’s perception	4.09 (2.81)	4.59 (2.62)	3.30 (2.54)
Observer’s perception	3.93 (2.85)	4.08 (2.90)	3.18 (2.48)
Pair’s average perception	4.00 (2.71)	4.31 (2.55)	3.24 (2.34)
Attitude toward money at stake – mean (SD) (0 = I don’t care at all; 8 = I care a lot)			
Player’s attitude	3.89 (2.08)	4.24 (2.56)	3.86 (2.58)
Observer’s attitude	4.44 (1.89)	4.22 (2.72)	3.88 (2.40)
Pair’s average attitude	4.17 (1.08)	4.23 (1.99)	3.87 (2.48)
Player as preferred role – %	77.8	67.3	74.1
Player’s preference	66.7	67.5	76.8
Observer’s preference	88.9	67.1	71.4
Expected to win – %	72.4	71.2	70.9
Player’s expectation	58.5	75.3	72.2
Observer’s expectation	86.4	67.1	69.6
Poker experience –mean (SD) (1 = never/rarely; 2 = sometimes; 3 = always)			
Player	1.24 (0.58)	1.20 (0.52)	1.18 (0.51)
Observer	1.30 (0.60)	1.14 (0.42)	1.25 (0.62)

In a broad, open-ended question, participants were also asked to explain the strategy they used to maximize their payoff (i.e., to win the game), but these responses were ultimately of little use: Players provided mostly intuitive answers (e.g., “I tried to display a neutral/identical facial expression”) whereas observers provided mostly intuitive, but vague answers (e.g., “I tried to observe the player’s facial expression”).

Finally, it is important to note that most of the data were collected in Brazil. In studies 1 and 2 supplementary data were gathered in the US to check for noticeable dissimilarities. No differences in the outcome of the game were observed. That being said, we suspect that region-specific cultural differences in emotion expression and/or beliefs could nevertheless emerge, which differences might subsequently affect the outcome of the game.

Future research could also address the role of learning (e.g., what would happen if participants were to play the game in multiple-rounds?), verbal signals (e.g., what would happen if players were asked to verbally state, truthfully or not, the content of the card?) and culture (e.g., what would happen if the game were played in a culture where neutral facial expressions are more prevalent?). These potential moderating variables, we suspect, could lead to meaningful, interesting changes in the outcome of game and give us broader insight into the role of contextual factors and strategic displays of facial emotion expressions in dyadic interactions.

## Author Contributions

FP: substantial contributions to the conception and design of the work; procedure design; data collection; data analysis. Revising the work critically for important intellectual content. Final approval of the version to be published. Agreement to be accountable for all aspects of the work in ensuring that questions related to the accuracy or integrity of any part of the work are appropriately investigated and resolved. EA: substantial contributions to the conception and design of the work; procedure design; data analysis. Drafting and revising the work critically for important intellectual content. Final approval of the version to be published. Agreement to be accountable for all aspects of the work in ensuring that questions related to the accuracy or integrity of any part of the work are appropriately investigated and resolved. PP: substantial contributions to the conception and design of the work; procedure design; data analysis. Revising the work critically for important intellectual content. Final approval of the version to be published. Agreement to be accountable for all aspects of the work in ensuring that questions related to the accuracy or integrity of any part of the work are appropriately investigated and resolved. SR: substantial contributions to data collection; data analysis. Revising the work critically for important intellectual content. Final approval of the version to be published. Agreement to be accountable for all aspects of the work in ensuring that questions related to the accuracy or integrity of any part of the work are appropriately investigated and resolved.

## Conflict of Interest Statement

The authors declare that the research was conducted in the absence of any commercial or financial relationships that could be construed as a potential conflict of interest.

## Footnotes

1 The label “player” rather than “sender” was used in the procedure and from now on it will be used throughout the remainder of the text.
