# Supplementary material for: What a Smile Means: Contextual Beliefs and Facial Emotion Expressions in a Non-verbal Zero-Sum Game
Source: Front Psychol. 2016 Apr 19;7:534. doi: 10.3389/fpsyg.2016.00534 (PMC4836202; doi:10.3389/fpsyg.2016.00534)
Supplement: Supplementary file 1 [file Data_Sheet_1.DOCX]

***Supplementary Material***

**What a smile means: contextual beliefs and facial emotion expressions in a nonverbal zero-sum game**

Fábio P. Pádua Jr., Paulo H. M. Prado ^*^, Scott S. Roeder, Eduardo B. Andrade

*** Correspondence:** Paulo H. M. Prado: pprado@ufpr.br

1. **Supplementary Data**

Since participants must “stare” at each other in a competitive setting, this game could be slightly uncomfortable when played among strangers. As a consequence, throughout all of the studies, care has been taken to perform each experimental session in an environment where most participants were of similar age *and* at least acquaintances (i.e., classmates).

See the procedure below provided to participants.

**-----------------**

**General Rules and Objectives of the Game *[USED IN ALL THREE STUDIES]***

**(Please read carefully)**

In this game, two participants are randomly paired and assigned to play the role of either the PLAYER or the OBSERVER. Two cards are distributed per pair. The cards must stay face down on the table, next to one another. One of the cards has a “0” sign whereas the other has a “$” sign printed on it. They are worth $0 and $10, respectively. The purpose of the game is to assess the extent to which the OBSERVER is capable of guessing which of the 2 cards has a $ on it simply by observing the PLAYER’S facial expression while/after s/he looks at each of the 2 cards. After the PLAYER has looked at both cards, the OBSERVER chooses one of them. If the OBSERVER selects the $ card, s/he ends the game with $10 and the PLAYER ends the game with $0. If the OBSERVER selects the 0 card, s/he ends the game with $0 and the PLAYER ends the game with $10.

Now, see below the exact sequence of steps during the game:

**Sequence of Steps of the Game**

- - 1. **PLAYER:** After the experimenter’s instructions, the PLAYER should:
       1. Take the first card from the table;
       2. Look to see if the card is printed with a “0” or a “$” sign;
       3. Look at the OBSERVER (eye-to-eye) for a few seconds;
       4. Return the card to the same place on the table;
       5. Take note (on the PLAYER’S sheet) of the actual value of the card ($0 vs. $10);
       6. Repeat the same process for the second card.
    2. **OBSERVER:** After the experiment’s instructions, the OBSERVER should:
       1. Wait until PLAYER takes card 1, looks at it, and makes eye contact with the OBSERVER;
       2. Then, indicate (on the OBSERVER’S sheet) his/her initial guess on whether the PLAYER has seen a $10 worth card or a $0 worth card.
       3. Repeat the same process after the PLAYER looks at the second card.
       4. Indicate (on the OBSERVER’S sheet) which Card s/he believes has the $ sign on it—that is, which card is worth $10.

**To BOTH participants:**

1. **The PLAYER should never let the OBSERVER see the sign on any of the cards, neither should the PLAYER let the OBSERVER know what the PLAYER wrote on the PLAYER’S sheet.**
2. **Any verbal communication is strictly prohibited.**
3. **At the end of the game (after the last card), the PLAYER should NOT reveal the cards. Likewise, the OBSERVER should NOT reveal his/her choice.**
4. **At the end of the game (after the last card), both participants will be asked to fill out a questionnaire.**
5. **After the final questionnaire, participants will be able to see the outcome of the game. Both participants should wait for additional instructions from the experimenter.**

**2 TRIAL CARDS**

To test your understanding of the game and its steps, you will now play a trial version with Cards Y and Z. No money is involved in this trial. After you finish the trial, the PLAYER should not reveal the cards NOR should the OBSERVER reveal their preference. The main purpose of the trial is to simply get the participants acquainted with the procedure. Once the game is finished, you will receive further instructions.

**CONSENT**

I, ______________________________________________, have read the instructions above and agree to participate in the experiment. Also, I declare I did not have participated in this study before and I have not participated in other experiments of this nature in the past 6 months.

_______________________________________________________________

Participant signature

THANK YOU AND GOOD LUCK!

**PLAYER’S SHEET *[USED IN ALL 3 STUDIES]***

2 TRIAL Cards

*Please indicate the card value ($10 or $0) after seeing each trial card*

| **Card Y** | **Card Z** |
| --- | --- |
| ( ) U$10 | ( ) U$10 |
| ( ) U$0 | ( ) U$0 |

**Now let’s start the actual game. There will be 2 actual cards (Cards 1 & 2).**

2 ACTUAL Cards

*Please indicate the card value ($10 or $0) after seeing each actual card*

| **Card 1** | **Card 2** |
| --- | --- |
| ( ) U$10 | ( ) U$10 |
| ( ) U$0 | ( ) U$0 |

**OBSERVER’S SHEET *[USED IN STUDIES 1 AND 2]***

2 TRIAL Rounds

*Please make your general assessment at the end of each trial round:**

| **Card Y** | **Card Z** |
| --- | --- |
| ( ) I’m certain this is the U$10 Card | ( ) I’m certain this is the U$10 Card |
| ( ) I think this is the U$10 Card | ( ) I think this is the U$10 Card |
| ( ) I have no idea | ( ) I have no idea |
| ( ) I think this is the U$0 Card | ( ) I think this is the U$0 Card |
| ( ) I’m certain this is the U$0 Card | ( ) I’m certain this is the U$0 Card |

**Now let’s play the actual rounds. There will be 2 rounds (Cards 1 & 2).**

2 ACTUAL Rounds

*Please make your general assessment at the end of each actual round:*

| **Card 1** | **Card 2** |
| --- | --- |
| ( ) I’m certain this is the U$10 Card | ( ) I’m certain this is the U$10 Card |
| ( ) I think this is the U$10 Card | ( ) I think this is the U$10 Card |
| ( ) I have no idea | ( ) I have no idea |
| ( ) I think this is the U$0 Card | ( ) I think this is the U$0 Card |
| ( ) I’m certain this is the U$0 Card | ( ) I’m certain this is the U$0 Card |

**AT THE END OF THE SECOND ACTUAL ROUND (AFTER CARD 2), please indicate which of the cards you believe has the $10 printed on it. If you guess correctly, you will receive the $10. If you get it wrong, your partner will receive the $10. ONLY ONE CHOICE IS ALLOWED.**

( ) Card 1 ( ) Card 2

**The main purposes of the general assessments were to (a) force participants to form an impression based on the facial expression after each card and (b) keep the flow of the game constant (e.g., player looks at card 1, puts it back on the table, observer grabs a pen from the table, indicates impression, puts pen back on the table, player then grabs the pen, indicates whether card 1 is $ or 0, puts pen back on the table, repeat). Note that only the final choice matters and observers are aware of it.*

**MEASUREMENTS USED IN STUDY 2 AND 3 RIGHT AFTER THE GAME.**

**PLAYER**

**YOUR FACIAL EXPRESSIONS**

**Now, please answer the questions below about YOUR OWN facial expressions AFTER you’ve seen Cards 1 and 2 (i.e., the actual Cards)**

**AFTER CARD 1**

1. Indicate on the scale below how serious or smiley your facial expression was after seeing Card 1:

| **Very Serious** |  |  | **Neutral** |  |  | **Very Smiley** |
| --- | --- | --- | --- | --- | --- | --- |
| -3 | -2 | -1 | 0 | 1 | 2 | 3 |

1. Indicate on the scale below how genuine or false your facial expression was after seeing Card 1**:**

| **Clearly False** |  |  | **Can’t tell** |  |  | **Clearly Genuine** |
| --- | --- | --- | --- | --- | --- | --- |
| -3 | -2 | -1 | 0 | 1 | 2 | 3 |

**AFTER CARD 2**

1. Indicate on the scale below how serious or smiley your facial expression was after seeing **Card 2:**

| **Very Serious** |  |  | **Neutral** |  |  | **Very Smiley** |
| --- | --- | --- | --- | --- | --- | --- |
| -3 | -2 | -1 | 0 | 1 | 2 | 3 |

1. Indicate on the scale below how genuine or false your facial expression was after seeing **Card 2:**

| **Clearly False** |  |  | **Can’t tell** |  |  | **Clearly Genuine** |
| --- | --- | --- | --- | --- | --- | --- |
| -3 | -2 | -1 | 0 | 1 | 2 | 3 |

**OBSERVER**

**YOUR PARTNER’S FACIAL EXPRESSIONS**

**Now, please answer the questions below about YOUR PARTNER’S facial expressions AFTER s/he saw Cards 1 and 2 (i.e., the actual Cards)**

**AFTER CARD 1**

1. Indicate on the scale below how serious or smiley your partner’s facial expression was after seeing **Card 1**:

| **Very Serious** |  |  | **Neutral** |  |  | **Very Smiley** |
| --- | --- | --- | --- | --- | --- | --- |
| -3 | -2 | -1 | 0 | 1 | 2 | 3 |

1. Indicate on the scale below how genuine or false your partner’s facial expression was after seeing **Card 1:**

| **Clearly False** |  |  | **Can’t tell** |  |  | **Clearly Genuine** |
| --- | --- | --- | --- | --- | --- | --- |
| -3 | -2 | -1 | 0 | 1 | 2 | 3 |

**AFTER CARD 2**

1. Indicate on the scale below how serious or smiley your partner’s facial expression was after seeing **Card 2:**

| **Very Serious** |  |  | **Neutral** |  |  | **Very Smiley** |
| --- | --- | --- | --- | --- | --- | --- |
| -3 | -2 | -1 | 0 | 1 | 2 | 3 |

1. Indicate on the scale below how genuine or false your partner’s facial expression was after seeing **Card 2:**

| **Clearly False** |  |  | **Can’t tell** |  |  | **Clearly Genuine** |
| --- | --- | --- | --- | --- | --- | --- |
| -3 | -2 | -1 | 0 | 1 | 2 | 3 |

**OBSERVER’S SHEET *[USED INSTUDY 3 - MEASUREMENT/MANIPULATION OF BELIEFS]***

2 TRIAL Rounds

*Please make your general assessment at the end of each trial round:*

| **Card Y** | **Card Z** |
| --- | --- |
| ( ) I’m certain this is the U$10 Card | ( ) I’m certain this is the U$10 Card |
| ( ) I think this is the U$10 Card | ( ) I think this is the U$10 Card |
| ( ) I have no idea | ( ) I have no idea |
| ( ) I think this is the U$0 Card | ( ) I think this is the U$0 Card |
| ( ) I’m certain this is the U$0 Card | ( ) I’m certain this is the U$0 Card |

-----------------------------------------------------------------------------------------------------------------------------------------

Before the actual rounds start, please answer the following questions: *

1) In your opinion, what does it mean when a player smiles while/after looking at a given card?

___A) It means s/he saw card ‘0” ___B) It means s/he saw card “$” **Correct Answer:** B [A]

2) In your opinion, what does it mean when a player displays a serious expression while/after looking at a given card?

___A) It means s/he saw card ‘0” ___B) It means s/he saw card “$” **Correct Answer:** A [B]

3) In your opinion, what does it mean when a player maintains a neutral expression while/after looking at a given card?

___A) It means s/he saw card ‘0” ___B) It means s/he saw card “$” **Correct Answer:** A or B

**-----------------------------------------------------------------------------------------------------------------------------------------**

**Now let’s play the actual rounds. There will be 2 rounds (Cards 1 & 2).**

2 ACTUAL Rounds

*Please make your general assessment at the end of each actual round:*

| **Card 1** | **Card 2** |
| --- | --- |
| ( ) I’m certain this is the U$10 Card | ( ) I’m certain this is the U$10 Card |
| ( ) I think this is the U$10 Card | ( ) I think this is the U$10 Card |
| ( ) I have no idea | ( ) I have no idea |
| ( ) I think this is the U$0 Card | ( ) I think this is the U$0 Card |
| ( ) I’m certain this is the U$0 Card | ( ) I’m certain this is the U$0 Card |

**AT THE END OF THE SECOND ACTUAL ROUND (AFTER CARD 2), please indicate which of the cards you believe has the $10 printed on it. If you guess it correctly, you will receive the $10. If you get it wrong, your partner will receive the $10. ONLY ONE CHOICE IS ALLOWED.**

( ) Card 1 ( ) Card 2

**Participants were randomly assigned to either the congruent belief induction (i.e., correct answers B and A to questions 1 and 2, respectively) or to the incongruent belief induction (i.e., correct answers A and B to questions 1 and 2, respectively). Question 3 did not include any manipulation. It was included simply to make sure participants would differentiate serious from neutral expressions.*
